# Taking Affective Learning in Digital Education One Step Further: Trainees’ Affective Characteristics Predicting Multicontextual Pre-training Transfer Intention

**DOI:** 10.3389/fpsyg.2020.02189

**Published:** 2020-09-17

**Authors:** Laurent Testers, Andreas Gegenfurtner, Saskia Brand-Gruwel

**Affiliations:** ^1^Breda University of Applied Sciences, Breda, Netherlands; ^2^Faculty of Arts and Humanities, University of Passau, Passau, Germany; ^3^Faculty of Educational Sciences/Open University of the Netherlands, Heerlen, Netherlands

**Keywords:** affective learning, distance education, training, transfer of learning, multicontextual transfer, intention to transfer, information literacy

## Abstract

The past decades have shown an accelerated development of technology-enhanced or digital education. Although an important and recognized precondition for study success, still little attention has been paid to examining how an affective learning climate can be fostered in online training programs. Besides gaining insight into the dynamics of affective learning itself it is of vital importance to know what predicts trainees’ intention to transfer new knowledge and skills to other contexts. The present study investigated the influence of five affective learner characteristics from the transfer literature (learner readiness, motivation to learn, expected positive outcomes, expected negative outcomes, personal capacity) on trainees’ pre-training transfer intention. Participants were 366 adult students enrolled in an online course in information literacy in a distance learning environment. As information literacy is a generic competence, applicable in various contexts, we developed a novel multicontextual transfer perspective and investigated within one single study the influence of the abovementioned variables on pre-training transfer intention for both the students’ Study and Work contexts. The hypothesized model has been tested using structural equation modeling. The results showed that motivation to learn, expected positive personal outcomes, and learner readiness were the strongest predictors. Results also indicated the benefits of gaining pre-training insight into the specific characteristics of multiple transfer contexts, especially when education in generic competences is involved. Instructional designers might enhance study success by taking affective transfer elements and multicontextuality into account when designing digital education.

## Introduction

This study, that took place within a distance learning environment, investigated to what extent five affective trainee characteristics influenced the students’ pre-training intention to transfer new learning from an information literacy course to two contexts: their study and their work.

One of the major developments in the field of education over the last decades has been the digitization of education. Due to the development of educational technologies, we have witnessed the emergence of a variety of forms of, and tools for interactive, collaborative and personalized learning. Terms that are used to describe these new environments are, amongst others, web-based, blended, digital, online, and distance learning environments. This development not only offers opportunities to widen access to education but also to design new learning spaces and develop and use new digital and interactive tools to optimize the educational experience and effectiveness. To optimally use and take advantage of these achievements a deeper insight into the learning processes and learners’ experiences within these digital environments is needed.

Our study was situated at the open university in the Netherlands, an institute that evolved from distance learning with paper study materials in the 1980s to an educational institute with personalized and activating online education. According to the hierarchy of Brindley et al. (2004) *online learning* is considered a subset of the overarching concept of *distance education*, characterized by the geographical separation between teachers and learners. As this gap was gradually regarded as a pedagogic shortcoming, the potential of online media to support and transform both teaching and learning in a variety of ways offered a means to bridge this separation (Bernard et al., 2004; Cunningham, 2017). Distance education distinguishes two major forms of instruction namely synchronous and asynchronous, although various blends exist also with face-to-face instruction. Synchronous means that students in a group are engaged in learning at the same time, much like the traditional face-to-face classroom, but not necessarily at the same place. Asynchronous education, having its roots in the traditional correspondence education, is individually based and time, place, and group independent. Respondents in our study, interchangeably referred to as students, learners, or trainees, participated in asynchronous learning with no direct physical or electronic contact with fellow students and mainly mail contact with their lecturers about their training assignments and results.

A recognized tool to design effective learning is Bloom’s Taxonomy. It offers a hierarchical set of learning objectives in three domains: the cognitive domain including mental skills or knowledge, the sensory domain encompassing manual or physical skills, and the affective domain referring to feelings, attitudes, and emotions. In this study we have focused on the affective domain reflecting the learner’s attitude toward the educational experience. This includes individual psychological aspects like attitudes, feelings, motivations, emotions, and values (Krathwohl et al., 1964). To facilitate learning in a face-to-face but also in an online learning environment it is considered important to foster a positive and motivating affective learning climate (Mazer et al., 2007) resulting in positive attitudes toward the training content, and lecturers and students who are feeling ready, able and motivated to participate in and successfully complete training. This also accounts for the subsequent step in the learning process namely the transfer of learning. Often interchangeably used with transfer of training, in this study it is more generally defined as the application of what has been learned to new situations (Testers et al., 2019). It has been studied for more than a century and is considered the *raison d’être* of education and an important indicator of the quality and success of the instructional design. On the other hand, research suggests that transfer of learning, especially in formal educational settings, is not self-evident. This paradox, also known as the transfer problem (Baldwin and Ford, 1988; Haskell, 2001), not only affects the quality of the education but also offers a poor return on investments in education. Baldwin and Ford (1988) have distinguished three domains of variables that might affect the transfer process: learner characteristics, training characteristics, and the organizational environment. A number of variables in the learner domain, including the ones that are used in our study, have an affective character and correspond with the individual aspects in the affective domain in Bloom’s Taxonomy: motivation or willingness to learn, expectations about the outcomes of the learning process and the personal capacity, and psychological readiness to participate. While research on transfer recognizes the importance of affective learner characteristics for the transfer process (Huang et al., 2015; Leberman and McDonald, 2016) little is known about how to foster affective learning (Boelens et al., 2017; Gegenfurtner et al., 2019) and an affective transfer climate in distance education environments. And even less information exists about affective predictors of the learners’ intention to transfer new learning and how to enhance their positive influence on the transfer process. Grounded in conceptual models in the transfer of training literature (Noe, 1986; Baldwin and Ford, 1988; Holton, 1996; Quesada-Pallarès and Gegenfurtner, 2015), this study intends to contribute to filling this gap and associate with a call for more research on the trainees’ perception of the learning context and their personal experiences (Baldwin et al., 2017). It investigated to what extent the students’ pre-training intention to transfer learning was influenced by five affective trainee characteristics.

### Intention to Transfer

The best way to investigate the effect of a specific variable on the transfer process would be to look at the resulting transfer. Besides the fact that there is no consensus on when to speak of a successful transfer, certain circumstances might hamper this assessment. One can think of transfer of so-called *open skills*, as opposed to closed skills, of which the application is not uniform and depends largely on the specific context and needs, and the learners’ creativity. Information literacy, the course the participants in this study were about to take, consisted of such open, complex higher-order cognitive skills (Brand-Gruwel et al., 2005; Reece, 2007). Furthermore, monitoring transfer might be problematic when it involves relative autonomous workers like the participants in this study. For these reasons, this study investigated the influence of five variables not on actual transfer, but on the students’ pre-training intention to transfer.

Although often used interchangeably, in this study the concepts motivation and intention are considered successive steps in a motivational process (Al-Eisa et al., 2009; Quesada-Pallarès and Gegenfurtner, 2015) where the intention to transfer intermediates between motivation to transfer and transfer itself. Motives explain why people act in a specific way while Ajzen in his Theory of Planned Behavior Ajzen (1991) considers intentions to capture these motives and subsequently indicate ‘how hard people are willing to try, how much of an effort they are planning to exert to perform the behavior. As a general rule, the stronger the intention to engage in a behavior, the more likely should be its performance’ (Ajzen, 1991, p. 181). According to Gollwitzer(1993, p. 147) “forming intentions is functional in the sense that it helps to achieve respective outcomes and to perform relevant behaviors.” Also the Goal-Setting Theory (Locke and Latham, 1990) and the Theory of Interpersonal Behavior (Triandis, 1980) consider intention a reliable predictor of behavior, including the transfer of training (Hutchins et al., 2013). Literature reviews show that the relationship between transfer variables and the intention to transfer new learning is largely missing from the literature (Cheng and Hampson, 2008; Hutchins et al., 2013). This study aims at contributing to filling this gap by investigating the influence of five independent variables on the dependent variable intention to transfer: learner readiness to transfer, motivation to learn, expected positive personal outcomes, expected negative personal outcomes, and personal capacity. It offers initial suggestions on how to enhance a positive affective transfer climate as an impetus to the design of strategies that are attuned to the specific study and work related conditions of their trainees.

### Learner Readiness

Learner or intervention readiness can be defined as the extent to which trainees are psychologically ready to enter and participate in training. As a rule, one can say that a positive pre-training perception of the program will enhance the learner’s preparedness to participate.

Research shows that learner readiness is a significant predictor of transfer of training and task performance (Ryman and Biersner, 1975; Hicks and Klimoski, 1987; Baldwin and Magjuka, 1991; Tannenbaum et al., 1991; Bates et al., 2007; Devos et al., 2007; Kulik et al., 2007; Bhatti et al., 2013). There is also evidence that learner readiness indirectly predicts transfer of training via its influence on the trainee’s motivation to learn (Sanders and Yanouzas, 1983; Holton, 1996; Knowles et al., 1998).

Previous studies on transfer suggest various affective individual characteristics that may enhance or impede the learners’ readiness and subsequently their transfer of training. It is for example important that a training program meets the learners’ individual needs and expectations and is relevant to their performance. Other aspects that are mentioned are for example training reputation and the expectations about its quality. Maurer et al. (2003) noticed that prior participation in training was a predictor of the trainees’ intention to participate in training.

Looking at these aspects learner readiness might be enhanced by involving learners already before the training in the instructional design process, for example by assessing their specific expectations and needs. This becomes more relevant but also more challenging in a globalizing world (Gegenfurtner et al., 2009) with the internationalization of education, and a tendency toward the personalization of learning in blended learning environments. This not only refers to the learners’ diverse backgrounds or learning preferences. Baharim (2008) for example found that learner readiness significantly differed across age, where older trainees (>41 years.) showed more readiness to participate in training than younger ones. He suggested that they might target the training more toward their career development requirements than younger trainees. Pre-training framing might not only prove a useful tool to enhance the trainees’ readiness to learn (Bates and Holton, 2004; Tai, 2006) but indirectly also their motivation to transfer (Ruona et al., 2002; Kirwan and Birchall, 2006; Devos et al., 2007). Instructional designers might, for example, offer a realistic preview of the training design, content end requirements and give learners a realistic impression of how the training will benefit their performance, in this case in their study and work context. Baldwin and Magjuka (1991) concluded that this, amongst others, leads to learners who have greater intentions to transfer and apply what they have learned back to their respective job settings.

In our study, the construct learner readiness has been operationalized in terms of the degree to which trainees are familiar with the training content, know how the training will improve their skills, and how it relates to their educational and professional development.

### Motivation to Learn

The most important precondition for transfer of training is actual learning; without learning there will be nothing to apply. This makes the motivation to learn, also referred to as pre-training motivation, not only an important aspect of affective learning but also a pre-condition for transfer. In the literature motivation to learn may refer to a general desire to enrich one’s knowledge and skills, and the consecutive aspiration to attend specific training. With a focus on the latter, we have defined motivation to learn as “the direction, intensity, and persistence of learning-directed behavior in training contexts” (Kanfer, 1991).

Extended research confirms that a learner’s motivation preceding training is a critical precursor not only to cognitive and skill-based training outcomes but also to transfer motivation and to transfer itself (Noe, 1986; Tannenbaum et al., 1991; Facteau et al., 1995; Quinones, 1995; Chiaburu and Marinova, 2005; Tziner et al., 2007). Various intrinsic and extrinsic individual and situational characteristics have been mentioned as predictors of training motivation, for example, self-efficacy, job involvement, learner readiness, familiarity with the training content and expected outcomes and utility (Jackson, 2014), age and work environment (Noe and Schmitt, 1986; Baldwin and Magjuka, 1991; Mathieu et al., 1992; Facteau et al., 1995; Kontoghiorghes, 2002). Also, the status of training is considered an important predictor. Although we learn from previous studies that attending on their own volition enhances the trainees’ motivation to learn (Hicks and Klimoski, 1987; Baldwin and Magjuka, 1991; Mathieu et al., 1992; Clark et al., 1993) also a mandatory training might increase training motivation (Baldwin and Magjuka, 1991; Rynes and Rosen, 1995; Cotterchio et al., 1998; Tsai and Tai, 2003; Gegenfurtner et al., 2016) when mandatory is considered an expression of the relative importance of the training to an organization or if attitudes toward the training, based on pre-training hearsay or personal experiences (Facteau et al., 1995), are very favorable. Additionally, Baldwin and Magjuka (1991) point to the degree of choice of training content as an important variable, rather than the choice of attending.

In our survey, we have measured the students’ pre-training motivation to participate in the training by asking them to what extent they consider this training important for their study and work, if they expect that the training will improve their performance in both contexts, and if not attending would feel like a missed opportunity.

### Expected Positive and Negative Personal Outcomes

Personal outcomes are the personal consequences of specific behavior, in this case, the application of new knowledge and skills to new situations. These outcomes can be positive as well as negative. Building on Rouiller and Goldstein (1993) and Vroom (1964) effort-performance and performance-outcome perceptions as causes of behavior Holton et al. (1997, 2000) defined positive personal outcomes as “the degree to which applying training on the job leads to outcomes that are positive to the individual.” These outcomes may include intrinsic and extrinsic incentives like increased personal satisfaction and growth opportunities (Facteau et al., 1995), increased productivity and work effectiveness, additional respect, salary increase or other rewards, the opportunity to further career development or to advance in the organization (Bates et al., 2012), verbal praise and bonuses (Xiao, 1996), higher performance evaluations (Facteau et al., 1995), and increased job security (Cheng, 2000). Positive personal outcomes are considered a significant predictor of perceived training transfer (Clarke, 2002; Ruona et al., 2002; Bates et al., 2007), of the learners’ motivation to learn (Noe, 1986; Facteau et al., 1995; Cheng, 2000), and of their motivation to transfer (Ruona et al., 2002; Nijman, 2004). According to Holton et al. (2000) supervisor support “serves as a reward to employees by signaling to them that their learning application efforts are viewed positively.” Perceiving appraisal support by supervisors and also peers before and after training, be it informational, instrumental or emotional, will enhance a belief in the relevance and applicability of the training and in the opportunities to apply new learning which, in turn, might lead to higher transfer outcomes (Nijman, 2004). In our pre-training study, we asked students if they think they should receive positive reactions when applying new learning, and if this should lead to rewards and positive performance evaluations, both in their study and work context.

Negative personal outcomes are the negative consequences for trainees of using (Rouiller and Goldstein, 1993) or not using learned knowledge and skills. In our study, we have focused on the latter and defined negative outcomes as the extent to which individuals believe that not applying skills and knowledge learned in training will lead to negative outcomes (Holton et al., 2000). These negative consequences, by Rouiller and Goldstein (1993) labeled as punishment and negative or no feedback, might include reprimands when not using new knowledge or skills on the job, penalties, peer resentment, reassignment to undesirable jobs, or reduced opportunities for a further job or career development or salary raises (Khasawneh et al., 2006). The limited research that is available on this construct (Nijman, 2004; Katsioloudes, 2015) shows that peer and supervisor support can be strong predictors of negative personal outcomes; the stronger the pre- and post-training support by peers and supervisors, the stronger their negative reactions for not using new learning (Nijman, 2004). While at the same time these negative reactions by peers or supervisors may increase the learners’ motivation to transfer (Ruona et al., 2002). In our survey, we have asked students if they think that not applying new learning will result in negative responses, negative performance evaluations, and criticism.

### Personal Capacity

In their Learning Transfer System Inventory (LTSI) Holton et al. (2000) address the ability to apply learning to the job by two elements: personal capacity and opportunity to use. Personal capacity is defined as the “extent to which individuals have the time, energy and mental space in their work lives to make changes required to transfer learning to the job” (Holton et al., 2000, p. 344). Studies using the LTSI model suggest that this construct is a significant predictor of transfer of training (Bates et al., 2007; Frash et al., 2010). This model also indicates an indirect influence on the transfer of training via motivation to transfer. Kirwan and Birchall (2006) underline the importance of both personal capacity and motivation for the realization of two key characteristics of transfer namely generalization and maintenance.

Looking at attributes associated with personal capacity a lack of time has been found to inhibit the transfer of new learning (Awoniyi et al., 2002; Clarke, 2002; Cromwell and Kolb, 2004; Gilpin-Jackson and Bushe, 2007) while low workload pressure was positively correlated to transfer (Awoniyi et al., 2002). Aspects of personal capacity may have been labeled differently for example as workload (Russ-Eft, 2002), work schedule, personal energy, and stress level (Bates and Holton, 2004), self-management (Richman-Hirsch, 2001), and dealing with situational constraints (Olivero et al., 1997). Also, variables at an individual level like age and gender may affect personal capacity (Velada et al., 2009).

Not surprisingly there is a strong relationship between personal capacity and the given opportunity to perform or apply new learning within a specific educational or organizational context in terms of “adequate equipment, information, human and financial resources, materials, and supplies” (Holton et al., 2007, p. 394). Within our study we have operationalized personal capacity by asking learners to what extent they had other obligations or life events that might prevent them from attending the training as intended, and to what extent they expected that work pressure and a lack of time might prevent them from practicing their newly gained competences. In the pre-training context of our study no learning and therefore no actual transfer had yet taken place. Research however shows that during and after learning personal capacity, that is closely related to Ajzen’s perceived behavioral control, may not only influence transfer via intention but can also mediate the relationship between intention and actual transfer.

In our study transfer of training, and thereby also the development of the intention to transfer, is considered a process that does not only takes place during a post-training test but that starts already before and also continues after an intervention (Baldwin et al., 2009; Sitzmann and Weinhardt, 2018). Education typically focuses on transfer at one point in time, mostly directly after training when the students’ knowledge, comprehension, and retention are tested (Blume et al., 2010). Educational designers also tend to concentrate predominantly on the training program when designing training for transfer. Research, however, shows that already before entering training specific conditions might enhance or inhibit the students’ transfer of training (Holton et al., 2000; Naquin and Holton, 2003). To complement previous research this study has asked students which affective trainee characteristics from the transfer literature already before the course influenced their intention to transfer prospective learning.

Furthermore, transfer of learning is generally measured within one specific context, mostly education or work. The distance learning students who participated in this research were studying beside their educational work and were starting a course in information literacy. This generic competence is not only useful in the context of their study but also in their educational work context. Our study, therefore, extends previous research by investigating the students’ intention to transfer to both their study and work context in one study.

## Research Question and Hypotheses

This study aimed at supporting the design of digital educational interventions that enhance the transfer of learning to multiple contexts by estimating the extent to which five affective learner characteristics predicted intention to transfer: learner readiness, motivation to learn, expected positive personal outcomes, expected negative personal outcomes, and personal capacity. Complementing previous literature (Testers et al., 2019) the present study adds a new aspect to transfer research by comparing two different transfer contexts within one single study: Study and Work.

This resulted in the central question: How are affective learner characteristics from the transfer literature associated with the intention to transfer training to the participants’ study and work contexts? We hypothesized positive relationships of learner readiness (Hypothesis 1), motivation to learn (Hypothesis 2), positive personal outcomes (Hypothesis 3), negative personal outcomes (Hypothesis 4), and personal capacity (Hypothesis 5) on the intention to transfer. No hypotheses were formulated on the expected differences in these relationships for the Study and the Work contexts as very limited previous research exists on multicontextual transfer.

## Materials and Methods

### Participants

In this study, we have questioned 366 adult students in their first year of the premaster Learning Sciences at the Open University of the Netherlands. Most of the students were teaching in primary, secondary and higher education and studied at the Open University beside their work. Table 1 shows the demographic characteristics of the participants, more specifically their gender, age, years of work experience, and work type. Differences in variable scores between female and male participants were statistically non-significant (*p* > 0.05).

**TABLE 1:** Demographic characteristics of the study participants.

Variable	Frequency	Percentage
Gender		
Female	284	77.60
Male	82	22.40
Age		
<25 years	70	19.13
25–35 years	119	32.51
36–45 years	100	27.32
46–55 years	64	17.49
56–65 years	13	3.55
Work experience		
<2 years	61	16.67
2–5 years	76	20.77
6–10 years	100	27.32
>10 years	129	35.25
Work type		
Permanent position	277	75.68
Temporary position	47	12.84
Temporary employment agency	18	4.92
Freelancer	14	3.83
Voluntary work	10	2.73

*Total sample size is N = 366.*

### Training Program and Procedure

To prepare them for their study, the participants were about to start a mandatory web-based course *Information Literacy for Social Scientists* (4,3 ECTS) (Wopereis et al., 2016). This training program was based on the Four-Component Instructional Design (4C/ID) model (Van Merriënboer and Kirschner, 2018), which included five authentic tasks each with varying a level of support (Brand-Gruwel et al., 2009), and performance feedback. One of the tasks was: “Write a blog post of about 400 words about the article that you have critically studied. Write a summary of 200 words and a critical examination of 200 words. In doing so use the guidelines for paraphrasing, citing, and referring correctly.” After the students were informed about the aim and content of the program but before they started with their first task, students filled out a questionnaire that was integrated into the electronic course as Task 0. Before taking the survey all students were informed that their responses would be used exclusively for this research and that their personal data and responses would be treated confidentially and with utmost care.

### Measures

A multi-item web-based online survey was used to collect the data. The authors used a novel design in which each question was related to both the participants’ Study and Work environment (Testers et al., 2019). The questionnaire used a 7-point Likert scale, ranging from 1 (*do not agree at all*) to 7 (*totally agree*). The dependent variable was the intention to transfer while the five independent variables were learner readiness, motivation to learn, positive personal outcomes, negative personal outcomes, and personal capacity. For reasons of comparability, the same number of variables and items were used for both the Study and Work context. Table 2 shows per scale the number of items, the reliability coefficient (Cronbach’s Alpha), and an example item for both the Study and the Work context.

**TABLE 2:** Number of items, reliability estimates, and example items of all scales.

Scales	Items	α	Example
**Dependent variable**			
Intention to transfer	3 (study)	0.94 (study)	I intend to apply the newly gained competences in my study
	3 (work)	0.96 (work)	I intend to apply the newly gained competences in my work
**Independent variables**			
Learner readiness	3 (study)	0.79 (study)	Prior to this course I know how the program is supposed to affect my information literacy in my study
	3 (work)	0.81 (work)	Prior to this course I know how the program is supposed to affect my information literacy in my work
Motivation to learn	3 (study)	0.82 (study)	I attend the course because I think it will improve my performance in my study
	3 (work)	0.77 (work)	I attend the course because I think it will improve my performance in my work
Personal outcomes pos.	3 (study)	0.73 (study)	I should receive positive reactions if I apply the newly gained competences from this course in my study
	3 (work)	0.83 (work)	I should receive positive reactions if I apply the newly gained competences from this course in my work
Personal outcomes neg.	3 (study)	0.82 (study)	I expect to be criticized if I do not utilize the newly gained competences in my study
	3 (work)	0.84 (work)	I expect to be criticized if I do not utilize the newly gained competences in my work
Personal capacity	3 (study)	0.87 (study)	At the moment there are other commitments or events in my life that prevent me from doing this course the way it should be done.
	3 (work)	0.92 (work)	

### Data Analysis

Initial screening of the data (cf. Kline, 2015) showed linearity, heteroscedasticity, univariate and multivariate normality, and no multivariate outliers. Missing data appeared to be missing at random and was treated with EM imputation (Allison, 2003). With several exploratory factor analyses (ML extraction, Oblimin rotation) the structure of the items of all six constructs was investigated separately for the Study and Work context. To achieve a clear and unambiguous structure several items from the original item set were removed (cf. Thurstone, 1947). Tables 3, 4 show the final results with the same six factors for both transfer contexts. In both contexts, the total variance explained confirms the utility of the model: 76.01% for the Study context and 76.52% for the Work context.

**TABLE 3:** Factor loadings of all scales in the transfer contexts *Study* and *Work*.

	Transfer context: *Study*						Transfer context: *Work*					
	Factor 1	Factor 2	Factor 3	Factor 4	Factor 5	Factor 6	Factor 1	Factor 2	Factor 3	Factor 4	Factor 5	Factor 6
Learner readiness	–0.014	0.023	0.708	0.037	–0.002	–0.053	0.038	–0.021	0.033	0.662	–0.161	0.013
	–0.029	–0.040	0.862	–0.034	–0.002	0.057	–0.038	–0.003	–0.006	0.824	0.155	–0.026
	0.120	0.009	0.623	0.065	0.104	–0.020	0.104	0.037	–0.055	0.711	0.167	0.053
Motivation to learn	0.019	–0.028	0.037	0.015	0.759	–0.016	0.090	–0.019	0.027	0.070	0.804	0.072
	–0.053	0.029	–0.001	–0.041	0.800	–0.014	0.159	0.017	–0.080	0.173	0.706	0.085
	0.103	–0.014	0.052	0.056	0.680	0.059	–0.013	–0.019	0.071	–0.064	0.501	0.008
Personal outcomes positive	–0.069	0.009	0.036	–0.046	–0.041	0.801	–0.076	0.060	–0.007	0.012	–0.027	0.804
	0.089	0.000	–0.091	0.167	0.205	0.460	0.084	–0.001	0.003	0.023	0.160	0.704
	0.108	0.023	–0.038	0.070	0.010	0.646	0.071	0.005	0.125	–0.007	0.004	0.721
Personal outcomes negative	–0.013	0.035	–0.010	0.785	0.034	–0.065	0.061	0.036	0.833	0.023	0.032	–0.028
	0.008	0.016	0.020	0.843	–0.059	0.115	0.072	0.062	0.888	–0.032	0.036	–0.030
	–0.003	–0.044	0.044	0.698	0.008	0.011	–0.032	–0.090	0.579	0.008	–0.015	0.155
Personal capacity	–0.022	0.612	0.135	–0.090	0.003	0.084	–0.147	0.412	0.142	0.076	0.087	–0.072
	0.006	0.905	–0.061	0.064	–0.005	–0.019	0.035	0.896	–0.076	–0.014	–0.057	0.123
	0.004	0.884	–0.095	0.042	0.003	–0.054	0.114	0.944	–0.064	–0.075	–0.073	0.056
Transfer intentions	0.960	–0.011	0.046	0.018	–0.045	–0.033	0.905	–0.033	0.067	0.079	0.031	–0.017
	0.819	–0.047	–0.057	–0.039	0.125	0.054	0.861	0.001	0.031	–0.047	0.115	0.059
	0.965	0.030	0.036	–0.004	–0.042	0.003	0.919	0.004	0.016	0.064	–0.016	–0.006

**TABLE 4:** Explained total variance of factors in the transfer contexts *Study* and *Work*.

	Transfer context					
	*Study*			*Work*		
Factor	Eigenvalue	% of variance	Cumulated%	Eigenvalue	% of variance	Cumulated %
1	5.19	28.81	28.81	5.71	28.90	28.90
2	2.59	14.38	43.19	2.43	11.14	40.03
3	2.13	11.82	55.01	2.01	11.09	51.13
4	1.50	8.33	63.34	1.71	7.76	58.89
5	1.16	6.45	69.79	1.17	4.40	63.29
6	1.12	6.22	76.01	0.95	4.23	76.52

**FIGURE 1:**
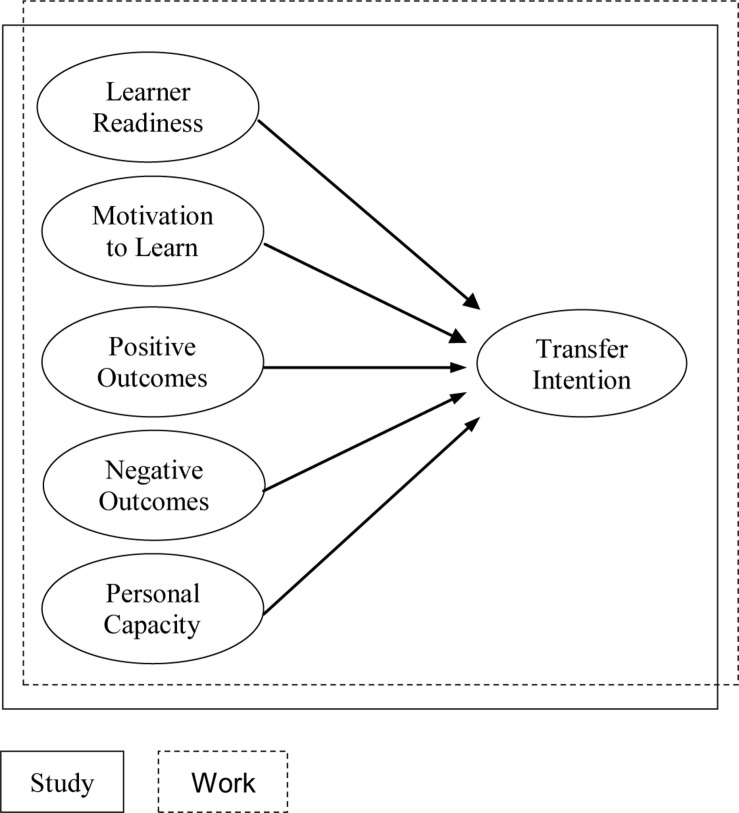
Hypothesized relationships in the transfer contexts *Study* and *Work*.

Structural Equation Modeling (EQS version 6.3) was used to test the model of Figure 1. For both the Study and Work context a “hybrid MRA model” was used, incorporating a confirmatory factor analysis, and a MRA model for measuring direct causal effects. To measure to what extent the hypothesized model fitted the research data five goodness-of-fit indices were used: χ^2^ to measure absolute fit, Comparative Fit Index (CFI), Incremental Fit Index (IFI), Standardized Root-Mean Square Residual (SRMR), and the Root-Mean Square Error of Approximation (RMSEA). In line with the recommendations of Hu and Bentler (1999) the cut-off criteria for acceptable model fit were: CFI >0.95, IFI >0.95, SRMR <0.08, and RMSEA <0.06.

## Results

The study intended to investigate to what extent the trainees’ pre-training intention to transfer was influenced by learner readiness (Hypothesis 1), motivation to learn (Hypothesis 2), positive personal outcomes (Hypothesis 3), negative personal outcomes (Hypothesis 4), and personal capacity (Hypothesis 5) in both their Study and Work context. Table 5 presents the means, standard deviations, reliability estimates, and intercorrelations amongst all six constructs.

**TABLE 5:** Correlation matrix of all variables.

	M	SD	1	2	3	4	5	6	7	8	9	10	11	12
**Transfer context: *Study***														
1. Learner readiness	4.75	1.48	(0.79)											
2. Motivation to learn	5.70	1.26	0.44^∗∗^	(0.82)										
3. Personal outcomes positive	5.15	1.51	–0.03	0.35^∗∗^	(0.73)									
4. Personal outcomes negative	4.86	1.87	0.12^∗^	0.42^∗∗^	0.45^∗∗^	(0.82)								
5. Personal capacity	2.89	1.65	–0.53	–0.06^∗∗^	0.11^∗^	0.00	(0.82)							
6. Transfer intention	6.53	0.84	0.24^∗∗^	0.57^∗∗^	0.36^∗∗^	0.42^∗∗^	–0.21^∗∗^	(0.94)						
**Transfer context: *Work***														
7. Learner readiness	4.19	1.48	0.79^∗∗^	0.34^∗∗^	–0.05	0.09	−0.02^∗^	0.15^∗^	(0.81)					
8. Motivation to learn	3.91	1.83	−0.12^∗^	–0.46^∗∗^	−0.13^∗^	–0.07	0.01	–0.1^∗∗^	–0.32^∗∗^	(0.77)				
9. Personal outcomes positive	4.23	1.82	0.05	0.27^∗∗^	0.47^∗∗^	0.19^∗∗^	0.10	0.17^∗∗^	0.18^∗∗^	–0.47^∗∗^	(0.83)			
10. Personal outcomes negative	2.30	1.45	0.03	0.15^∗^	0.23^∗∗^	0.20^∗∗^	0.16^∗∗^	–0.04	0.11^∗^	–0.46^∗∗^	0.44^∗∗^	(0.84)		
11. Personal capacity	3.24	1.84	0.07	0.13^∗^	0.17^∗∗^	0.14^∗^	0.61^∗∗^	–0.05	0.04	0.03^∗^	0.24^∗∗^	0.11	(0.78)	
12. Transfer intention	4.96	1.77	0.27^∗∗^	0.36^∗∗^	0.06	0.04	–0.06	0.31^∗∗^	0.47^∗∗^	–0.45^∗∗^	0.55^∗∗^	0.16^∗∗^	0.08	(0.96)

*Cronbach’s alpha estimates in brackets on the diagonal. ^∗^p < 0.05, ^∗∗^p < 0.01.*

In both contexts the six-factor model generated an acceptable fit. Table 6 shows the psychometric properties. In the Study context the *X*^2^ was 207.69 (*df* = 120), CFI = 0.97, IFI = 0.97, SRMR = 0.05, and RMSEA = 0.05 (90% CI = 0.04, 0.06). In the transfer context Work, the *X*^2^ was 252.34 (*df* = 120), CFI = 0.96, IFI = 0.96, SRMR = 0.05, and RMSEA = 0.06 (90% CI = 0.05, 0.07). These estimates suggest an acceptable and comparable model fit for both contexts.

**TABLE 6:** Goodness-of-fit indices of the structural models in the transfer contexts *Study* and *Work*.

	Transfer context	
	*Study*	*Work*
*X*^2^ (df)	207.96 (120)	252.34 (120)
CFI	0.97	0.96
IFI	0.97	0.96
SRMR	0.05	0.05
RMSEA (90% CI)	0.05 (0.04; 0.06)	0.06 (0.05; 0.07)

**FIGURE 2:**
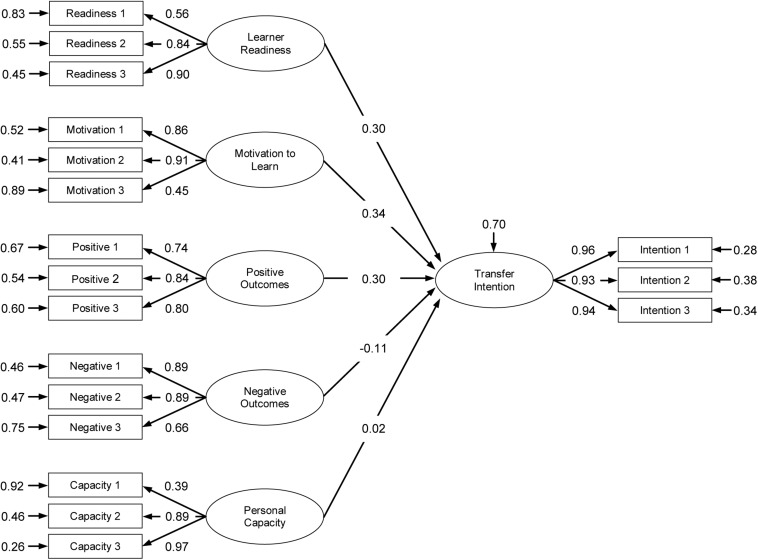
Measurement and structural model parameter estimates of transfer context: Study.

**FIGURE 3:**
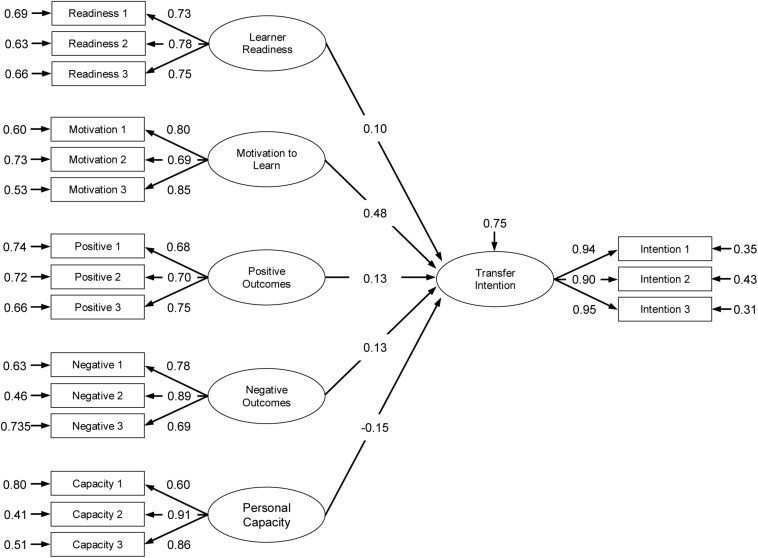
Measurement and structural model parameter estimates of transfer context: Work.

The model parameter estimates of the structural relations among factors for the Study and Work contexts are presented in Figures 2, 3. In the transfer context Study, intention to transfer was positively predicted by motivation to learn (β = 0.48, *p* < 0.01), personal outcomes positive (β = 0.13, *p* < 0.01), personal outcomes negative (β = 0.13, *p* < 0.01), and learner readiness (β = 0.10, *p* < 0.01); the relationship between personal capacity and intention to transfer (β = −0.15) was statistically non-significant. In the transfer context Work, intention to transfer was predicted by motivation to learn (β = 0.34, *p* < 0.01), personal outcomes positive (β = 0.30, *p* < 0.01), and learner readiness (β = 0.30, *p* < 0.01); the relationship of intention to transfer with personal capacity (β = 0.02) and personal outcomes negative (β = −0.11) were statistically non-significant.

A comparison between the two transfer contexts Study and Work showed different model parameter estimates between the independent and dependent variables. Table 7 presents the differences between beta coefficients for all variables. The highest difference emerged for Personal Outcomes Negative (Study context: β = 0.13, Work context: β = −0.11, Δ = 0.24) followed by Learner Readiness (Study context: β = 0.10, Work context: β = 0.30, Δ = 0.20), Personal Capacity (Study context: β = −0.15, Work context: β = 0.02, Δ = 0.17) and Personal Outcomes Positive (Study context: β = 0.13, Work context: β = 0.30, Δ = 0.17), and finally Motivation to Learn (Study context: β = 0.48, Work context: β = 0.34, Δ = 0.14). These analyses tend to indicate the benefits of examining multiple transfer contexts when estimating learners’ characteristics predictors of intention to transfer.

**TABLE 7:** Comparison of beta coefficients between the two transfer contexts *Study and Work*.

	Transfer context		
Influence on intention to transfer	Study	Work	Δ
Learner readiness	0.10	0.30	0.20
Motivation to learn	0.48	0.34	0.14
Personal outcomes positive	0.13	0.30	0.17
Personal outcomes negative	0.13	-0.11	0.24
Personal capacity	-0.15	0.02	0.17

## Discussion

This study has explored learning processes in digital education, more specifically how to foster an affective learning climate in a distance education environment that enhances the learners’ intention to transfer new learning We consider the transfer or application of new learning a key aspect of learning processes and an essential indicator of study success. Complementing sparse research on the intention to transfer learning within distance education settings we have investigated trainees’ pre-training perceptions of the importance of five affective trainee characteristics for their intention to transfer new learning to their Study and their Work context.

Previous literature indicates that transfer of learning in educational settings is not only happening during post-training tests. It is a process that is influenced by a variety of factors not only during but also before and after a training program. The first finding of our pre-training study confirms that already before the actual training several variables, in this case, five affective learner characteristics may influence the learners’ intention to transfer new learning. Educational designers might take this into account when creating affective learning climates that facilitate the transfer of new learning.

The second finding of our study is that there is a difference between the beta coefficients for both transfer contexts. This indicates that a multicontextual perspective might be appropriate when designing education of generic competences for transfer. Looking at the relative importance of the five variables to the trainees’ intention to transfer new learning we noticed that in the Study context motivation to learn (β = 0.48, Hypothesis 2), personal outcomes positive (β = 0.13, Hypothesis 3), personal outcomes negative (β = 0.13, Hypothesis 4), and learner readiness (β = 0.10, Hypothesis 1) positively predicted transfer intention while its relationship with personal capacity (β = −0.15, Hypothesis 5) was non-significant. In the transfer context Work we found that intention to transfer was significantly predicted by motivation to learn (β = 0.34, Hypothesis 2), personal outcomes positive (β = 0.30, Hypothesis 3), and learner readiness (β = 0.30, Hypothesis 1) while personal capacity (β = 0.02, Hypothesis 5), and personal outcomes negative (β = −0.11, Hypothesis 4) were statistically non-significant. These findings indicate that transfer enhancing or impeding conditions within these contexts may differ and that it might prove beneficial for educational designers to adopt a multicontextual perspective, especially when it involves generic competences like information literacy that can be used in, or are specifically meant for multiple transfer contexts. Course designers might inventory and discuss these context-specific transfer conditions during the training and stimulate trainees’ reflections on how to create and foster their personal optimal affective transfer climate.

Looking at the results in more detail we see that motivation to learn was the strongest predictor of transfer intention in both contexts, although stronger in the Study than the Work context. This might not come as a surprise as the framing of the training, mandatory and given at the very beginning of the premaster, might be considered an indication that this training must be important to the students’ study success, which in turn will enhance their motivation to learn. The results also confirm findings from previous studies where the learners’ pre-training motivation to learn is considered a critical precursor not only of cognitive and skills-related learning outcomes but also of transfer motivation and in effect of transfer itself (Noe, 1986; Tannenbaum et al., 1991; Facteau et al., 1995; Quinones, 1995; Chiaburu and Marinova, 2005; Tziner et al., 2007). Without initial learning, there will be nothing to transfer. This makes the enhancement of this affective learner characteristic all the more important. From the literature, we learn that various intrinsic and extrinsic individual and situational factors may influence the trainees’ pre-training motivation to learn and to transfer, including age and work environment (Noe and Schmitt, 1986; Baldwin and Magjuka, 1991; Mathieu et al., 1992; Facteau et al., 1995; Kontoghiorghes, 2002; Jackson, 2014). In our study motivation to learn referred to the trainees’ motivation to participate in and learn from the specific training in information literacy they were about to take, not to their motivation to learn in general. To support the motivation process educational designers might involve trainees when designing the training content, for example by offering the opportunity for students to use cases from their daily practice for their assignments. It is also important to communicate before the start of the training the content and expected outcomes and their utility for contexts relevant to the students. This appears to be more important for adult learners, the participants in our research. Training that meets their specific needs for practical applicability not only predicts learning, as confirmed by adult learning theories (Wlodkowski and Ginsberg, 2017) but also transfer of learning (Leberman et al., 2006; Nafukho et al., 2017). The fact that this training is mandatory and situated at the very beginning of the trainees’ pre-master Learning Sciences could be presented as indicators of its importance. Future research might complement existing studies on the effect of voluntary and mandatory training participation on the transfer process (Gegenfurtner et al., 2016), including on motivation to learn and on the intention to transfer. Also, learner readiness, another affective learner characteristic from this study, proved to be a significant predictor of transfer intention, although more in the Work than in the Study context. This might be explained by the fact that in the Study context learner readiness should not be an issue as students who started the training were, or at least were supposed to be, ready to apply new learning in their assignments. In the Work context application of new learning was not self-evident or immediately required but more a personal choice, which would make feeling ready more relevant. Previous research shows that learner readiness might indirectly affect the actual transfer of training via its influence on motivation to learn (Holton, 1996; Sanders and Yanouzas, 1983; Knowles et al., 1998). Already before the actual start of the training learner readiness, and consequently transfer itself, can be enhanced in various ways. Training might focus on more generalizable principles that can be customized according to specific context requirements. Trainees can for example also be involved in the instructional design process, offering them opportunities to express their specific needs and expectations before and during the training. This would be in line with the contemporary attention for the learner’s uniqueness, resulting in a more personalized way of teaching and learning. Another way to enhance learner readiness, and also their motivation to learn and intention to transfer, would be pre-training framing. A positive pre-training perception of the program will enhance the trainees’ preparedness to attend the training. This can be achieved by offering a realistic preview of the training, for example by communicating what can be expected in terms of content, quality, and relevance to the trainees’ transfer contexts. Knowing what to expect and how they will be supported during the training might enhance the learners’ self-efficacy and thereby indirectly their readiness to attend the training.

Previous studies on transfer show that also expected positive and negative personal transfer outcomes may predict the trainees’ motivation to learn (Noe, 1986; Facteau et al., 1995; Cheng, 2000), as well as their motivation to transfer (Ruona et al., 2002; Nijman, 2004) and actual transfer (Clarke, 2002; Ruona et al., 2002; Bates et al., 2007). The respondents in our study considered expected positive personal outcomes relevant for their intention to transfer new learning in both their Study and Work context. This can be expected for their Study context as the proper use of new learning will lead to increased effectiveness and positive performance evaluations. But trainees considered positive outcomes more relevant in their Work context. This despite the fact that they worked relatively autonomous and that using new learning from this one specific training in their work might not be noticed by peers and supervisors. One explanation might be that, working mainly as lecturers and tutors, newly gained information literacy competencies might be considered useful for their educational activities as well as for their personal development, for example when searching for high-quality information to stay up-to-date in their profession. The effect of expected negative outcomes on the trainees’ intention to transfer was only significant in the trainees’ Study context. This seems obvious as not using new learning during the training will inevitably lead to negative feedback from lecturers or lower grades for assignments. Not applying new learning in the Work context will probably go unnoticed and will not lead to negative consequences like peer or supervisor resentment.

Finally, the variable personal capacity appeared to be not significant in both transfer contexts, indicating that the trainees didn’t expect that their intention to transfer new learning to their Study and Work contexts would be hampered by other obligations or a lack of time and energy to practice.

The initial recommendations in our study to facilitate a positive and motivating affective transfer climate in a distance learning environment are in line with general recommendations on how to enhance affective learning. They can be used as an impetus for complementary strategies aligned to the specific conditions of the training and the trainees. When designing a training program instructional designers might for example stimulate the trainees’ willingness or motivation to learn by giving them opportunities before and during the training to express their personal preferences and specific needs for their transfer context(s), by communicating the quality, relevance, and gains of attending the training, by encouraging, and by building self-efficacy. Literature confirms that peers and supervisors are important actors in building and maintaining an encouraging, inspiring and productive learning and transfer climate, not only during but also before the actual start of the training. During our research, the a-synchronous training the participants were about to take was characterized by the absence of physical and online contact between students while contact between students and lecturers was limited to reviewing and advising on the assignments. Social presence (Spears, 2012) and social interaction (Jung et al., 2002; Swan, 2003; Yang et al., 2010) are considered to be two important preconditions for successful online and affective learning (Sun and Chen, 2016; Richardson et al., 2017). After our study, the format of the training has been altered and now includes more synchronous interaction between students through collaborative group assignments. This however also requires more time and planning of the adult trainees who often have a multitude of obligations besides their study. Future research within this altered setting but also in more traditional face-to-face environments might show in what way social presence and social interaction influence the relationship between affective learner characteristics like learner readiness and motivation to learn, and the intention to transfer.

This study contributes to the conceptual development of affective learning by complementing limited previous research on variables that might enhance or impede an affective transfer climate in distance education environments. It also adds a new aspect to transfer theories by adopting a multicontextual perspective on transfer, investigating the transfer process to two application contexts within one sample. Results confirm the value of this new perspective and of the importance of affective learner characteristics to the transfer process in distance education.

The practical relevance of our study is that it advises educational designers on how to create and maintain an affective learning climate that enhances the transfer of new learning. They might, for example, take into account that transfer is not only the application of new learning during a post-training test but a process that is influenced by a multitude of variables already before the actual start of training. Our study more specifically underlines the importance of affective learner characteristics to the transfer process. Involving trainees in the program design and proper framing might enhance training transfer. It might also be useful to realize that if transfer, especially when it involves generic competences, is meant or suitable for use in multiple contexts these may each have their specific transfer enhancing or impeding conditions. Gaining insight into these contexts and discussing them during training might prove profitable to the transfer process.

There are several limitations to this study. The first limitation is the use of only a self-report survey for collecting the data. Although there are legitimate arguments against the use of only this one source, including the risk of common method bias, specific conditions might prevent the use of additional data sources like observation, interviews, psychological signal measurements, or actual transfer measurements. In our study, respondents were distance learners studying from home and working relatively autonomous at various locations throughout the Netherlands. Also, new learning from the training consisted of so-called open higher-order thinking skills that can be executed in a variety of ways. This makes monitoring and measuring the transfer process, including the development of transfer intention of the individual participants near to impossible. For future research, however, we would opt for triangulation of the data when circumstances allow. Furthermore, this study took place in a very specific educational environment, namely distance adult education. Future research might be expanded to face-to-face or blended learning environments and to pre-adult learners. And finally, the participants in our study were predominantly female (77.6%). In general, research findings on the effect of gender on training related variables tend to be inconsistent (Powell, 1988; Bass and Stogdill, 1990). This may be the result of different research settings like field or laboratory (Dobbins and Platz, 1986), and the research focus. Looking at variables that predict training outcomes, for example, Tziner and Falbe (1993) observed that gender affected the motivation to transfer. Velada et al. (2009) concluded that male respondents had higher perceptions of several training variables from the Learning Transfer System Inventory, including “positive personal outcomes” and “personal capacity to transfer” than female respondents. The present study has been focused on the relationship between pre-training transfer intentions and five trainee-related variables. Results show that differences in variable scores between female and male participants were statistically non-significant (*p* > 0.05). Future research might look closer into the role of demographic characteristics on the transfer process and in what way these characteristics are influenced by, for example, specific research, cultural, work, or educational conditions.

We have tried to minimize undesirable bias by emphasizing in advance that responses would be treated anonymously and with the greatest care and that the electronic survey offered the possibility to answer the questions in private.

Our study intended to extend limited research on the influence of affective learner characteristics on transfer processes in a-synchronous online distance education by adopting a pre-training and multicontextual perspective. Results indicate that these perspectives and the constructs and items used in this study may offer educational designers practical tools to design educational interventions that will enhance the learners’ intention to transfer new learning, and in the end transfer itself. We welcome future studies that confirm, challenge or complement the value of these perspectives for transfer research. In the next step of our research, we will investigate the temporal dimension of the transfer process by comparing the effects of individual, instructional and environmental predictors of intention to transfer before, directly after and 3 months after the training.

## Data Availability Statement

The datasets generated for this study are available on request to the corresponding author.

## Ethics Statement

Ethical review and approval was not required for the study on human participants in accordance with the local legislation and institutional requirements. Before the participants started with their first task, students filled out a questionnaire that was integrated into the electronic course as Task 0. Before taking the survey all students were informed that their responses would be used exclusively for this research and that their personal data and responses would be treated confidentially and with utmost care.

## Author Contributions

LT was involved in the acquisition of the data. AG and LT performed data analysis and interpretation. SB-G and AG critically revised the manuscript. All authors contributed substantially to the design and execution of the study and approved the final version.

## Conflict of Interest

The authors declare that the research was conducted in the absence of any commercial or financial relationships that could be construed as a potential conflict of interest.
